# The diagnosis and management of systemic autoimmune rheumatic disease-related interstitial lung disease: British Society for Rheumatology guideline scope

**DOI:** 10.1093/rap/rkae056

**Published:** 2024-04-18

**Authors:** Jennifer Hannah, Mia Rodziewicz, Puja Mehta, Kerri-Marie Heenan, Elizabeth Ball, Shaney Barratt, Sara Carty, Richard Conway, Caroline V Cotton, Sarah Cox, Anjali Crawshaw, Julie Dawson, Sujal Desai, Ahmed Fahim, Carol Fielding, Mark Garton, Peter M George, Harsha Gunawardena, Clive Kelly, Fasihul Khan, Gouri Koduri, Helen Morris, Marium Naqvi, Elizabeth Perry, Claire Riddell, Cristiana Sieiro Santos, Lisa G Spencer, Nazia Chaudhuri, Muhammad K Nisar

**Affiliations:** Academic Rheumatology, Faculty of Life Sciences and Medicine, King’s College London, London, UK; Centre for Musculoskeletal Research, University of Manchester, Manchester, UK; Centre for Inflammation and Tissue Repair, University College London, London, UK; Department of Respiratory Medicine, Northern Health and Social Care Trust, Antrim, UK; Department of Rheumatology, Belfast Health and Social Care Trust, Belfast, UK; Department of Respiratory Medicine, Bristol Medical School, Bristol, UK; Department of Rheumatology, Great Western Hospitals NHS Foundation Trust, Swindon, UK; Department of Rheumatology, Trinity College Dublin, Dublin, Ireland; Department of Rheumatology, Liverpool University Hospitals NHS Foundation Trust, Liverpool, UK; Patient Representative, London, UK; Department of Respiratory Medicine, University Hospitals Birmingham NHS Foundation Trust, Birmingham, UK; Department of Rheumatology, St Helens Hospital, Saint Helens, UK; Radiology Department, Royal Brompton Hospital, London, UK; Department of Respiratory Medicine, New Cross Hospital, Wolverhampton, UK; Patient Representative, London, UK; Department of Rheumatology, Royal Shrewsbury Hospital, Shrewsbury, UK; Department of Respiratory Medicine, Royal Brompton Hospital, London, UK; North Bristol NHS Trust, Academic Rheumatology, Bristol, UK; Department of Rheumatology, James Cook University Hospital, Middlesbrough, UK; Department of Respiratory Medicine, University Hospitals of Leicester NHS Trust, Leicester, UK; Department of Rheumatology, Southend University Hospital NHS Foundation Trust, Southend-on-Sea, Essex, UK; Department of Respiratory Medicine, Wythenshawe Hospital, Manchester, UK; Department of Respiratory Medicine, Guy’s and St Thomas’ Hospitals NHS Trust, London, UK; Department of Rheumatology, University Hospitals Bristol and Weston NHS Foundation Trust, Bristol, UK; Department of Rheumatology, Belfast Health and Social Care Trust, Belfast, UK; Department of Rheumatology, Complejo Asistencial Universitario de Leon, Leon, Spain; Department of Respiratory Medicine, Aintree University Hospital, Liverpool, UK; School of Medicine, University of Ulster, Coleraine, UK; Rheumatology Department, Luton, Dunstable University Hospital, Luton, UK

**Keywords:** guideline, interstitial lung disease, connective tissue disease, UK, scope

## Abstract

Interstitial lung disease (ILD) is a significant complication of many systemic autoimmune rheumatic diseases (SARDs), although the clinical presentation, severity and outlook may vary widely between individuals. Despite the prevalence, there are no specific guidelines addressing the issue of screening, diagnosis and management of ILD across this diverse group. Guidelines from the ACR and EULAR are expected, but there is a need for UK-specific guidelines that consider the framework of the UK National Health Service, local licensing and funding strategies. This article outlines the intended scope for the British Society for Rheumatology guideline on the diagnosis and management of SARD-ILD developed by the guideline working group. It specifically identifies the SARDs for consideration, alongside the overarching principles for which systematic review will be conducted. Expert consensus will be produced based on the most up-to-date available evidence for inclusion within the final guideline. Key issues to be addressed include recommendations for screening of ILD, identifying the methodology and frequency of monitoring and pharmacological and non-pharmacological management. The guideline will be developed according to methods and processes outlined in Creating Clinical Guidelines: British Society for Rheumatology Protocol version 5.1.

## Why the guideline is needed

Systemic autoimmune rheumatic diseases (SARDs) is a term that covers a broad spectrum of clinical conditions of autoimmune aetiology [1]. Interstitial lung disease (ILD) is a common manifestation of several SARDs and is associated with excess morbidity and mortality.

SARDs associated with ILD include RA, SSc, SS, idiopathic inflammatory myopathy (IIM), MCTD, SLE, ANCA-associated vasculitis (AAV) and axial SpA (axSpA). The terms CTD and collagen vascular disease have been historically used to describe this group of conditions and particularly SSc, SS, IIM, MCTD and SLE. However, CTD and collagen vascular disease are not reflective of our current understanding of the pathophysiology or organ involvement of these conditions and the term SARD is therefore preferred for the purposes of this guideline.

There are currently no UK guidelines related to the management of SARD-ILD and there is a concern that the condition may be underdiagnosed and consequently undertreated. It is important to understand that within the broad spectrum of SARD-ILD, distinct clinical phenotypes such as rapidly progressive ILD and progressive pulmonary fibrosis (PPF) (also termed progressive fibrosing ILD) have specific diagnostic and management considerations.

Strategies for screening people with existing SARDs for ILD are not clearly defined. In RA, many people have subclinical ILD and it is unclear if abnormalities on imaging in the absence of clinical symptoms should influence treatment and the frequency of monitoring. Screening for ILD also has the potential to overdiagnose minor interstitial lung abnormalities as ILD, the prognostic significance of which is unclear.

The data supporting the use of commonly used immunomodulatory strategies and newer therapies (e.g. antifibrotic drugs) in the treatment of SARD-ILD is steadily growing [2–4]. Antifibrotic drugs are now recommended in the National Institute for Health and Care Excellence (NICE) guideline for pulmonary fibrosis–ILD [5] and it is vital that all healthcare professionals caring for people with SARD-ILD are aware of the appropriate use of such treatment.

A EULAR task force is expected to publish its guideline for SARD-ILD in 2024 and the ACR published their summary guidance in August 2023 [6]. However, there is a need for UK-specific guidance that takes into account the resources of the UK health service, including local licencing and funding policies. The guideline will therefore be a useful document to support local and national policy-making in the UK.

This guideline will be developed using the methods and processes outlined in Creating Clinical Guidelines: British Society for Rheumatology Protocol [7].

## Facts and figures

SARDs are estimated to account for ≈30% of ILDs [8]. Among those with SARD, the incidence of ILD varies. Interstitial lung abnormalities in RA are common, but only 5–11% are thought to result in clinically significant symptoms [9, 10]. ILD is estimated to affect 39–72% with MCTD, 44–50% with SSc, 33–50% with IIM, 12–21% with primary SS [11], 3–40% with AAV [12–14], 3–10% with SLE [11] and 7–30% with axSpA [15, 16]. The outlook is highly variable following SARD-ILD diagnosis. Life expectancy can vary widely, e.g. from 8 years post-diagnosis in RA-ILD [17] to 6-month survival rates of 33–66% in those with rapidly progressive melanoma differentiation-associated protein 5–positive IIM-ILD [18].

## Current practice

Current practice is variable across the UK [19]. Most people with SARD-ILD are managed in secondary or tertiary care settings, with dual-specialty rheumatology–respiratory clinics and multidisciplinary teams (MDTs) operating in some, but not all, centres. Previous disease-specific British Society for Rheumatology (BSR) guidelines have advocated screening for ILD in all people with SSc [20] and those with high-risk IIM subtypes [21], but no such recommendations have been made in RA or other SARDs.

The NICE idiopathic pulmonary fibrosis (IPF) guideline states that people with suspected ILD should be referred to a specialist centre for confirmation of the diagnosis and development of a treatment plan by an MDT with relevant clinical expertise and training [22]. The approach to SARD-ILD diagnosis can vary depending on the availability of resources and expertise. Some medical therapies are used off-licence and reimbursement is often dependent on validation from the treating tertiary centre, but varies by region.

Immunosuppressive medication is frequently used in the treatment of SARD-ILD, including corticosteroids, azathioprine, mycophenolate mofetil, abatacept, tocilizumab, rituximab and cyclophosphamide [2, 3, 23–25]. Antifibrotic medications are now approved by NICE for use in people with progressive non-IPF ILD and may be used alongside immunomodulatory therapies [5].

In RA, methotrexate (MTX) is a recommended first-line treatment for active joint disease. However, it is still sometimes avoided in those with RA-ILD due to concerns regarding MTX pneumonitis, which carries a higher mortality in people with reduced pulmonary reserve [26]. This remains an area of controversy and will be examined in detail in the guideline.

## Who the guideline is for?

This guideline is for any health professional in the UK who cares directly for people with SARD-ILD, including rheumatologists, pulmonologists, rheumatology and respiratory specialist nurses, radiologists, primary care clinicians, allied health professionals, specialty trainees, pharmacists and other stakeholders such as patient organizations and charities.

## Equality considerations

People of all ethnicities are affected by SARD-ILD, meaning language and cultural barriers will have to be addressed to ensure all receive equitable access to care and high-quality education and treatment.

## What will the guideline cover?

This guideline is being developed to provide recommendations on the screening, diagnosis, monitoring, pharmacological and non-pharmacological management of adults with SARD-ILD in the UK.

## Groups that will be covered

Overarching principles will cover people with ILD associated with RA, SSc, SS, IIM, MCTD, SLE, AAV and axSpA. Another group included at the scoping stage are those people with ILD in whom clinical and serological profiles raise suspicion of SARD but do not meet classification criteria of a specific diagnosis. This will include patients in which ILD is the first, the predominant or the sole manifestation of SARD. This group remains poorly defined and previous labels include interstitial pneumonia with autoimmune features (IPAF), undifferentiated CTD-associated ILD, lung-dominant CTD and autoimmune-featured ILD. Each term has different but overlapping criteria. For the purposes of this guideline, this group will be referred to as ‘ILD with suspected SARD’ in order to ensure that our literature search includes relevant evidence for all the aforementioned terms.

**Figure 1.**
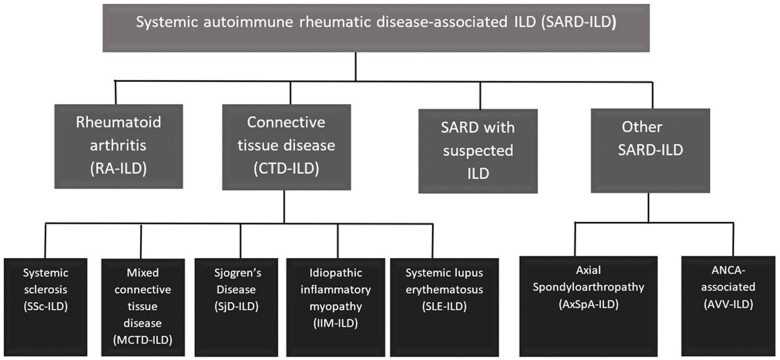
Chart demonstrating the types of SARD-ILD that will be included in the guideline. These have been grouped into four sections to aid the organization of the systematic review

For the purposes of the literature review, we elected to divide SARDs into the following four groups (Fig. 1): RA-ILD, CTD-ILD (SSc, IIM, MCTD, SS and SLE-related ILD), other (AAV and axSpA-related ILD) and ILD with suspected SARD. We acknowledge that these categories have limitation, and do not reflect clinical manifestations or the underlying pathophysiology of these conditions.

## Groups that will not be covered

This guideline is not intended to cover people with idiopathic pulmonary fibrosis or other causes of ILD such as asbestosis, silicosis, hypersensitivity pneumonitis, radiation or non-anti-rheumatic drug-induced ILD.

A statement on the management of pulmonary sarcoidosis has recently been published by the British Thoracic Society (BTS) [27], and while sarcoidosis is an important cause of ILD, management of its multisystem manifestations is beyond the scope of this guideline.

This guideline will not cover the diagnosis and management of SARD-ILD in paediatric populations (<16 years of age). ILD is a rare complication of SARD-ILD in children and there is insufficient literature available upon which to make any evidence-based recommendations. Also, the management of pulmonary hypertension and other complications will not be covered.

## Settings

The guideline is being developed primarily for use in rheumatology and respiratory secondary and tertiary care settings but will also be of interest to other specialities involved in shared care, including primary care.

### Related guidance

2023—ACR guideline for the screening and monitoring of interstitial lung disease in people with systemic autoimmune rheumatic disease (publication pending).
2008—Interstitial lung disease guideline: the British Thoracic Society in collaboration with the Thoracic Society of Australia and New Zealand and the Irish Thoracic Society [28].
2022—American Thoracic Society (ATS), European Respiratory Society (ERS), Japanese Respiratory Society (JRS), and Asociación Latinoamericana de Tórax (ALAT) guideline: idiopathic pulmonary fibrosis (an update) and progressive pulmonary fibrosis in adults: an official ATS/ERS/JRS/ALAT clinical practice guideline [29].
2021—Interstitial lung disease associated with autoimmune rheumatic diseases: checklists for clinical practice (Italian guideline—Delphi process) [30].
2020—Japanese 2020 guide for the diagnosis and treatment of interstitial lung disease associated with connective tissue disease [31].

RA-ILD:

2022—Spanish Societies of Rheumatology and Pneumology and Thoracic Surgery recommendations for the management of rheumatoid arthritis–related interstitial lung disease. Part 2: treatment [32].

SSc-ILD:

2017—Update of EULAR recommendations for the treatment of systemic sclerosis [33].

2023—Treatment of systemic sclerosis-associated interstitial lung disease: evidence-based recommendations. an official American Thoracic Society clinical practice guideline [34].

2016—BSR and British Health Professionals in Rheumatology guideline for the treatment of systemic sclerosis [20].

IIM-ILD:

2022—BSR guideline on management of paediatric, adolescent and adult patients with idiopathic inflammatory myopathy [21].

Sjögren’s-ILD:

2021—ACR consensus guidelines for evaluation and management of pulmonary disease in Sjögren’s [35].

Sarcoidosis:

2021—European Respiratory Society clinical practice guidelines on treatment of sarcoidosis [36].

2021—BTS clinical statement on pulmonary sarcoidosis [27].

2020—Diagnosis and detection of sarcoidosis. An official American Thoracic Society clinical practice guideline [37].

## Key issues and draft questions

The following key issues and draft questions have been identified by the working group and will be used to develop more detailed questions to be addressed following a systematic search of the literature.

- Which people with known SARD should be screened for ILD?
  - How often should screening take place?
  - What modalities should be used for screening?
  - How should subclinical ILD be managed when identified in people with SARD?
- What pharmacological management should be considered in SARD-ILD?
- What non-pharmacological therapies are of clinical benefit?
- Should any anti-rheumatic drugs be avoided or used with caution in those at risk of SARD-ILD?
- How should people newly diagnosed with ILD, with positive SARD-associated autoantibodies but no other clinical manifestations of a SARD be managed?

## Proposed guideline structure

### Overarching principles

Screening:

- Who should be screened? Does this differ according to underlying SARD or by antibody profile?
- What tests should be performed to screen for ILD?
  - Clinical examination
  - Chest X-ray
  - High-resolution computed tomography (HRCT) of the chest
  - Lung function tests
  - Six-minute walk test
  - Lung ultrasound
- How do we determine prognosis?
  - Autoantibody profile
  - Lung function test parameters
  - Imaging features on HRCT chest
  - Quantitative HRCT chest analyses
  - Functional exercise capacity tests
  - Soluble biomarkers
  - Patient reported outcome measures

Monitoring:

- What complications should we screen for?
  - Pulmonary hypertension (due to ILD)
  - Gastro-oesophageal reflux disease
  - Glucocorticoid-related complications
  - Progressive pulmonary fibrosis
- At what frequency and by which modes should people with SARD-ILD be monitored?
  - Frequency of lung function tests
  - Frequency of HRCT chest imaging
- How should we define deterioration and improvement?
  - When to escalate or de-escalate therapy

Management:

- How should management of the underlying SARD be balanced with the management of ILD?
- Are any anti-rheumatic drugs associated with the development or worsening existing SARD-ILD?
- How should those with ILD found to have positive autoantibodies without underlying SARD be managed?
- Specific interventions to be examined are:
  - Glucocorticoids—dose and route
  - Conventional synthetic DMARDs:
    - Mycophenolate mofetil
    - MTX
    - Leflunomide
    - Azathioprine
    - Calcineurin inhibitors
    - Cyclophosphamide
  - Biologic DMARDs:
    - Janus kinase inhibitors
    - Interleukin-6 inhibitors (e.g. tocilizumab)
    - TNF inhibitors
    - Abatacept
  - Anti-CD20 monoclonal antibodies (e.g. rituximab)
  - IVIG
  - Anti-fibrotic therapies
  - Proton pump inhibitors
  - Plasma exchange
  - Extra-corporeal membrane oxygenation
  - Lung transplant
  - Haematopoietic stem cell transplant
  - Physiotherapy and pulmonary rehabilitation
  - Antimicrobial prophylaxis

In order to produce evidence-based recommendations for specific treatments, a systematic literature search will be undertaken using the Population, Intervention, Comparison and Outcomes (PICO) framework.

### Population

The population includes patients with RA-ILD, CTD-ILD (SSc, IIM, MTD, SS and SLE), other SARD-ILD (AAV, axSpA) and ILD with suspected SARD.

### Comparison

Comparison will be against either placebo or standard-of-care therapy.

### Outcomes

The primary outcome to be examined will be progression as measured by the decline in pulmonary function tests. Secondary outcomes will include mortality, acute exacerbation rate, radiological progression, quality of life and potential serious adverse effects of the intervention.

Narrative/consensus recommendations will also be produced to cover the following aspects of management:

- Palliative care/symptomatic management
- Oxygen therapy
- Vaccinations
- Alternative therapies—hyperbaric oxygen therapy, essential oils, acupuncture, hypnotherapy, ayurveda, homeopathy, naturopathy, Chinese or Oriental medicine, dietary supplements
- Smoking
- Nutrition

## Approaches to audit of the guideline

Audit tools will be created to enable guideline users to easily assess local service delivery.

## Guideline working group

Muhammad Nisar (chair), consultant rheumatologist; Ahmed Fahim, consultant respiratory physician; Anjali Crawshaw, consultant respiratory physician; Carol Fielding, patient representative; Caroline Cotton, consultant rheumatologist; Claire Riddell, consultant rheumatologist; Clive Kelly, consultant rheumatologist; Cristiana Sieiro Santos, consultant rheumatologist; Elizabeth Perry, consultant rheumatologist; Fasihul Khan, consultant respiratory physician; Gouri Koduri, consultant rheumatologist; Harsha Gunawardena, consultant rheumatologist; Helen Morris, ILD specialist nurse; Jennifer Hannah, rheumatology specialist trainee; Julie Dawson, consultant rheumatologist; Kerri-Marie Heenan, respiratory specialist trainee; Lisa G. Spencer, consultant respiratory physician; Liz Ball, consultant rheumatologist; Marium Naqvi, highly specialist pharmacist; Mark Garton, consultant rheumatologist; Mia Rodziewicz, rheumatology specialist trainee; Nazia Chaudhuri, consultant respiratory physician; Peter George, consultant respiratory physician; Puja Mehta, rheumatology specialist trainee; Richard Conway, consultant rheumatologist; Sara Carty, consultant rheumatologist; Sarah Cox, patient representative; Shaney Barrett, consultant respiratory physician; Sujal Desai, consultant radiologist

## Data Availability

No new data were generated or analysed in support of this article.
