# Correction to: The diagnosis and management of systemic autoimmune rheumatic disease-related interstitial lung disease: British Society for Rheumatology guideline scope

**DOI:** 10.1093/rap/rkae085

**Published:** 2024-07-27

This is a correction to: Jennifer Hannah, Mia Rodziewicz, Puja Mehta, Kerri-Marie Heenan, Elizabeth Ball, Shaney Barratt, Sara Carty, Richard Conway, Caroline V Cotton, Sarah Cox, Anjali Crawshaw, Julie Dawson, Sujal Desai, Ahmed Fahim, Carol Fielding, Mark Garton, Peter M George, Harsha Gunawardena, Clive Kelly, Fasihul Khan, Gouri Koduri, Helen Morris, Marium Naqvi, Elizabeth Perry, Claire Riddell, Cristiana Sieiro Santos, Lisa G Spencer, Nazia Chaudhuri, Muhammad K Nisar, The diagnosis and management of systemic autoimmune rheumatic disease-related interstitial lung disease: British Society for Rheumatology guideline scope, *Rheumatology Advances in Practice*, Volume 8, Issue 2, 2024, rkae056, https://doi.org/10.1093/rap/rkae056

This paper has been updated to correct an error in author Peter M. George’s name.

